# Accuracy of Eye and Hair Color Prediction in Mexican Mestizos from Monterrey City Based on ForenSeq^TM^ DNA Signature Prep

**DOI:** 10.3390/genes14051120

**Published:** 2023-05-22

**Authors:** José Alonso Aguilar-Velázquez, Blanca Jeannete Llamas-de-Dios, Miranda Fabiola Córdova-Mercado, Carolina Elena Coronado-Ávila, Orlando Salas-Salas, Andrés López-Quintero, Benito Ramos-González, Héctor Rangel-Villalobos

**Affiliations:** 1 Instituto de Investigación en Ciencias Biomédicas, Centro Universitario de Ciencias de la Salud, Universidad de Guadalajara (CUCS-UdeG), Guadalajara 44340, Jalisco, Mexico; 2 Departamento de Morfología, Centro Universitario de Ciencias de la Salud, Universidad de Guadalajara (CUCS-UdeG), Guadalajara 44340, Jalisco, Mexico; 3 Licenciatura en Ciencias Forenses, Centro Universitario de Ciencias de la Salud, Universidad de Guadalajara (CUCS-UdeG), Guadalajara 44340, Jalisco, Mexico; blanca.llamas8863@alumnos.udg.mx; 4 Instituto de Criminalística y Servicios Periciales, Fiscalía General de Justicia del Estado de Nuevo León (FGJNL), Monterrey 64720, Nuevo León, Mexicocarolina.coronado@fiscalianl.gob.mx (C.E.C.-Á.); benito.ramos@fiscalianl.gob.mx (B.R.-G.); 5 Instituto de Nutrigenética y Nutrigenómica Traslacional, Centro Universitario de Ciencias de la Salud, Universidad de Guadalajara (CUCS-UdeG), Guadalajara 44340, Jalisco, Mexico; 6 Instituto de Investigación en Genética Molecular, Centro Universitario de la Ciénega, Universidad de Guadalajara (CUCI-UdeG), Ocotlán 47820, Jalisco, Mexico

**Keywords:** MPS, Forenseq, phenotypes, Mexico, genomics, piSNPs

## Abstract

Forensic genomic systems allow simultaneously analyzing identity informative (iiSNPs), ancestry informative (aiSNPs), and phenotype informative (piSNPs) genetic markers. Among these kits, the ForenSeq DNA Signature prep (Verogen) analyzes identity STRs and SNPs as well as 24 piSNPs from the HIrisPlex system to predict the hair and eye color. We report herein these 24 piSNPs in 88 samples from Monterrey City (Northeast, Mexico) based on the ForenSeq DNA Signature prep. Phenotypes were predicted by genotype results with both Universal Analysis Software (UAS) and the web tool of the Erasmus Medical Center (EMC). We observed predominantly brown eyes (96.5%) and black hair (75%) phenotypes, whereas blue eyes, and blond and red hair were not observed. Both UAS and EMC showed high performance in eye color prediction (*p* ≥ 96.6%), but a lower accuracy was observed for hair color prediction. Overall, UAS hair color predictions showed better performance and robustness than those obtained with the EMC web tool (when hair shade is excluded). Although we employed a threshold (*p* > 70%), we suggest using the EMC enhanced approach to avoid the exclusion of a high number of samples. Finally, although our results are helpful to employ these genomic tools to predict eye color, caution is suggested for hair color prediction in Latin American (admixed) populations such as those studied herein, principally when no black color is predicted.

## 1. Introduction

In the last several decades, short tandem repeat (STR) loci have become the golden standard in DNA forensic casework. Nevertheless, STR systems can be ineffectual in some situations as in low-quality or quantity samples, resulting in a missing or incomplete STR profile for human identification (HID) purposes. Single nucleotide polymorphisms (SNPs) have been targeted for analysis to address these forensic issues, given that the shorter amplicon length generated with SNPs increases the analytical success of degraded DNA samples [1]. Additionally, SNPs provide a research tool to infer some individual traits, such as physical appearance and biogeographic origin. This is relevant in the context of decedents whose remains may offer a poor visual indication of their physical features, such as in cases of advanced decomposition or mass disasters [2], especially when DNA profiles are absent from available databases [1]. Thus, information derived from phenotype-informative SNPs (piSNPs) and ancestry-informative SNPs (aiSNPs) can be essential in cold cases for the HID process [3].

The first practical tool to infer eye color phenotypes (IrisPlex) was developed in 2011 [4] and, subsequently, it was expanded to infer both eye and hair coloration (HIrisPlex) [5]. The system showed >94% prediction accuracy for blue and brown eye colors, while for intermediate eye color was 77%; for hair color, the average prediction accuracy for blond, brown, red, and black were 69.5, 78.5, 80, and 87.5%, respectively [5]. Massively parallel sequencing (MPS) is an emerging tool in forensic science that is particularly beneficial for overcoming the shortfalls of STR profiling [6,7]. This technology accomplishes the simultaneous analysis of STRs, identity-informative SNPs (iiSNPs), phenotype informative (piSNPs) [8], and ancestry informative (aiSNPs) [9], as well as mitochondrial DNA analysis [10,11]. Among the forensic MPS systems, the ForenSeq^TM^ DNA Signature Prep kit (Verogen, San Diego, CA, USA) contains two separated DNA primer sets: (1) DNA Primer Set-A (DPS-A), targets and amplifies 27 autosomal STRs (aSTRs), 7 X-chromosome STRs (X-STRs), 24 Y-chromosome STRs (Y-STRs), and 94 iiSNPs, and; (2) DNA Primer Set-B (DPS-B), targets all those markers included in DPS-A plus 56 aiSNPs and 22 piSNPs [12]. The piSNPs of the DPS-B are based on the markers included in the HIrisPlex.

The DPS-A of this forensic MPS system has been analyzed worldwide [13,14,15,16,17,18,19,20]. Conversely, scarce population studies based on DPS-B have been reported; they are limited to the Norwegian population [21], Yavapai ethnic group [22], and the four major ethnic groups from the USA [23]. To our best knowledge, this task has not been conducted in Latin American or Mestizo (admixed) populations, which is of great interest because of their complex population structure, resulting from ~500 years of admixture since the European colonization of the Americas. In the Mexican population, their major ancestral contributors involve Spaniards, Native Americans, and—to a lesser extent—African slaves. An ancestral gradient along the Mexican territory has been reported for Mestizos: the European ancestry shows a decreasing North-to-South gradient, whereas the Native American ancestry displays the contrary decreasing South-to-North pattern. Thus, the southeast of Mexico—also known as Mesoamerica in the pre-Columbian period—shows the highest Native American ancestry [24].

Although iris color prediction and ancestry inference has been carried out in European and South American populations [25], it is necessary to predict the accuracy of forensic MPS platforms in admixed populations with complex genetic structures. Particularly in Mexican Mestizos, where brown and black coloration predominates for both eye and hair phenotypes, respectively. In this paper, we assessed—for the first time in a Latin American population—the accuracy of eye and hair color based on the ForenSeq™ DNA Signature Prep DPS-B in the Mexican Mestizo population from Monterrey City (Northeast, Mexico), which is the second main economic, cultural, and political metropolis of this country.

## 2. Materials and Methods

### 2.1. Population Sample

Previously, we reported the forensic parameters of the genetic markers included in the DPS-A of 105 unrelated residents of Monterrey City in the Nuevo Leon state (Northeast, Mexico) [19]. In this study, most of these samples were analyzed with the DPS-B to infer eye and hair color (*n* = 88). Seventeen samples were excluded from the original sample set due to the impossibility of phenotype observation (individuals with dyed hair or baldness). For the 88 samples, reported information on the sample donor regarding hair color, eye color, birthplace, and ancestry was provided in the informed consent. All volunteers signed an informed consent form before their inclusion in the study, according to the Helsinki Declaration Ethical Guidelines. This work was approved by the Ethical Research Committee at the Institute of Criminalistics and Forensic Services of the Attorney General of the Nuevo Leon state (Project number assigned: IC-017-2019).

### 2.2. Eye and Hair Color Phenotype Observed

A high-resolution photograph was taken of all volunteers to establish confident eye and hair color phenotypes. For this purpose, we used a Canon Eos Rebel T6 camera. For qualitative determination of eye and hair colors, two individuals were separately asked to intuitively assign each photograph to one of the following phenotype categories: (1) eye color: blue, intermediate, or brown, where intermediate color was assigned to those neither blue nor brown; (2) hair color, according to the enhanced prediction method: black, dark brown/black, brown/dark brown, brown, dark blond/brown, blond/dark blond, blond, and red; and (3) hair color, according to the original HIrisPlex method: blond, red, brown, or black. The coincidence between interpreters to assign the volunteers’ phenotypes simplified to establish a final color designation in the total sample. The criteria of color categories were taken based on the IrisPlex and HIrisPlex models. We found predominantly brown and black colors for both eye and hair phenotypes as described below.

### 2.3. DNA Extraction Method

DNA was obtained from peripheral blood using the Prep-Filer Express BTA™ Forensic DNA extraction kit. For this purpose, the AutoMate Express DNA extraction system was used according to the supplier’s instructions (Applied Biosystems, Foster City, CA, USA). Next, DNA was quantified with the Quantifiler^®^ Trio DNA quantification kit in a 7500 Applied Biosystems Real-Time PCR system (Applied Biosystems, Foster City, CA, USA).

### 2.4. Massive Parallel Sequencing (MPS) Method

Libraries were generated using the DPS-B of the ForenSeq™ DNA Signature Prep kit (Verogen^®^, San Diego, CA, USA). Library preparation involved PCR to amplify the DNA targets (piSNPs and aiSNPs) and incorporate dual-indexed adaptors [12]. The libraries were normalized and then pooled. A total of 12–32 samples were pooled on each run. The library pool was diluted, denatured, and then added to the MiSeq FGx™ reagent standard and micro kits for cluster generation on the flow cell according to manufacturer recommendations. Sequencing was conducted following the procedures outlined in the MiSeq FGx™ Instrument Reference Guide [14]. After each sequencing run was completed, a post-run wash was performed. We carried out five sequencing runs, two of them with 12 samples on the Microflow cell and three runs with 32 samples on the Standard Flow cell, including positive and negative amplification controls in each run. However, 17 samples were excluded as mentioned above. Sequencing results were analyzed with the Universal Analysis Software v1.3 (UAS) provided by the manufacturer and using its default parameters for variant calling and then retrieved and downloaded in an Excel sheet.

### 2.5. Data Analysis

Genotype data generated by the MiSeq FGx™ instrument was processed and analyzed using Verogen’s Universal Analysis Software v1.3 (UAS). Then, genetic data were downloaded in Microsoft Excel format. Allelic frequencies, observed heterozygosity (Ho), expected heterozygosity (He), Hardy–Weinberg equilibrium (HWE), and linkage disequilibrium (LD) were evaluated with the Excel complement GenALEx v6.5 [26] and GDA v1.1 software [27]. The piSNP data were input into the Erasmus Medical Center (EMC) web tool (https://hirisplex.erasmusmc.nl (accessed on 20 May 2023), Rotterdam, Netherlands). The R script supplied by the EMC web tool was employed to convert the Excel piSNP format—provided by the UAS—to the specific format needed for the web tool. Although UAS and EMC predictors use multinomial logistic regression (MLR) prediction models, UAS was only trained with a European reference dataset based on the original HIrisPlex System [5]. Conversely, the EMC web tool provides an enhanced prediction model that considers hair color and shade, and it was trained with an extended dataset comprising individuals with various continental origins, as follows: 80% from Europe, 16% from North America (including individuals of European, African, and Asian ancestry), and 4% from Africa and Oceania [4,5,28]. Predictions obtained with the UAS and the EMC web tool were compared among each other, as well as with the color phenotypes based on the volunteer’s photographs. Both the UAS and the EMC web tool included blue, intermediate, and brown eye color inference providing *p*-values for each phenotype. For hair color inference, UAS produced the outcomes black, brown, red, and blond, whereas the EMC web tool produced different outcomes: black, dark brown/black, brown/dark brown, brown, dark blond/brown, blond/dark blond, blond, and red. For hair color estimation, we employed the following methods: (1) the enhanced prediction algorithm (HirisPlex-S DNA Phenotyping Webtool User Manual 2.0 (2018), found at: https://hirisplex.erasmusmc.nl accessed on 20 May 2023) for the EMC predictions considering the hair color and shade (black, dark brown/black, brown/dark brown, brown, dark blond/brown, blond/dark blond, blond, and red) and (2) employing only the black, brown, blond, and red categories for comparison purposes between the two algorithms. For comparative and assessment purposes, two different levels of predicted *p*-values were taken into account, as follows: (1) considering the highest *p*-value (pMax) without restriction: where no threshold was considered as long as prediction difference ≥10% between the two higher values (i.e., brown = 0.49, blond = 0.39, thus we assumed the brown color prediction), whereas the result was considered inconclusive when the difference between probabilities was <10%; and (2) employing a threshold of probabilities ≥70% (i.e., brown = 0.71, blond = 0.29, thus we assumed the prediction of brown color), while we declared inconclusive when the probability of the prediction was <70% (i.e., brown = 0.69, blond = 0.31).

## 3. Results

### 3.1. Genetic Population Data

Genotypes of 24 piSNPs from 88 samples are available in Supplementary Materials S1. Allelic frequencies, Ho, and HWE results are available in Table 1. The higher Ho was observed in rs885479 (Ho = 0.523), while rs312262906, rs1805006, rs11547464, rs201326893, and rs1805008 were in monomorphic state in the Monterrey population (Ho = 0). We observed two loci (rs1042602 and rs1800407) out of Hardy–Weinberg expectations (*p* > 0.05). However, when the Bonferroni correction was applied (*p* > 0.002), all loci were in HWE (Table 1). We found 19 pairs of loci in linkage disequilibrium (LD), in which 2 loci stand out, the rs1042602 (*TYR* gene) and rs1800407 loci (*OCA2* gene) because they were involved in 14 and 5 of these 19 pairwise LD cases, respectively. When Bonferroni correction was applied, only two pairs of loci were in LD, again both rs1042602 and rs1800407 (Supplementary Material S2). Due to the forensic purpose of this work, these findings will not be further discussed.

### 3.2. Eye color Prediction Performance

**Figure 1:**
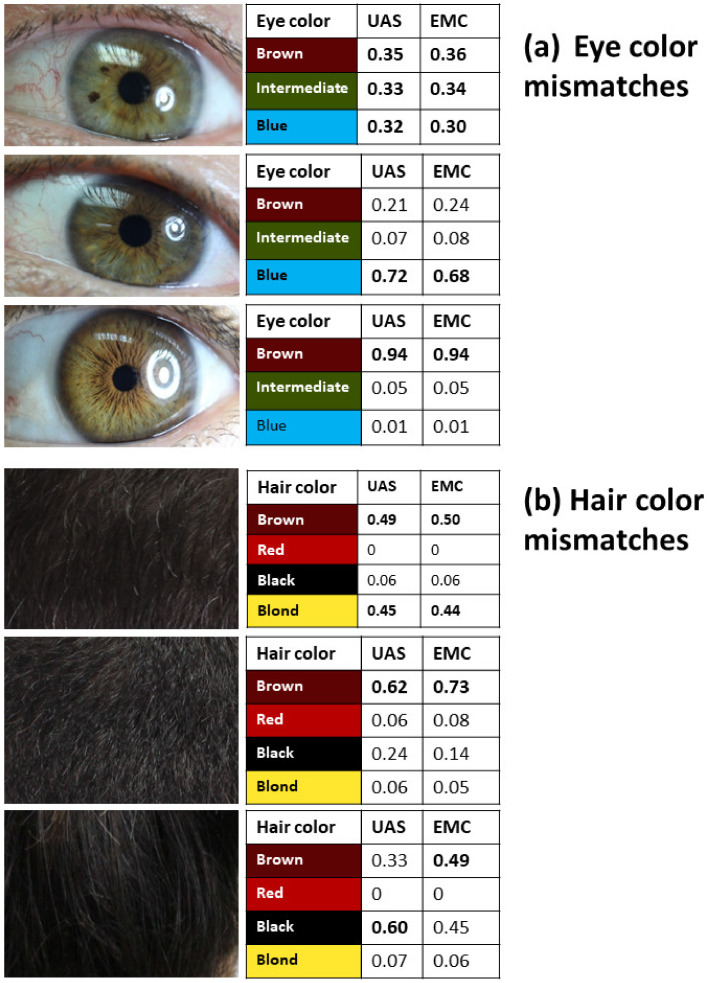
Mismatches between predicted and observed phenotypes for eye (**a**) and hair (**b**) colors in the Mestizo population sample from Monterrey City (Northeast, Mexico).

No dropouts were found in the sample set; therefore, all samples were predicted (*n* = 88) for both UAS and EMC systems. We observed eighty-five brown eyes (96.6%) and three intermediate eye colorations (3.4%) in the Monterrey City population, where no blue eyes were observed. On the other hand, the UAS and EMC systems without restrictions (pMax) predicted eighty-six brown eyes and one blue eye, plus one inconclusive result; nevertheless, only eighty-five brown eye assignments were correct (96.6%) (Table 2a). However, when we excluded those predictions with probabilities < 70% (three samples), the prediction increased to 98.8% for the two systems. Both UAS and EMC failed to predict accurately the three intermediate eye colors observed in the whole sample (Figure 1). In the first case, the observed eye color was green, but predictors results were inconclusive for both systems (*p* = ~0.3 for each color). The second case was hazelnut color, but predictors suggest blue color (*p* = 0.72 for UAS and *p* = 0.68 for EMC). For the last case, amber color was observed, but both predictors showed a brown eye color prediction (*p* = 0.94) (Figure 1). Although we reviewed the results of the principal component analysis (PCA) for these three samples, the same ancestry pattern of Mestizo individuals from northern Mexico was observed; thus, ancestry results did not provide additional relevant information.

### 3.3. Hair Color Prediction Performance

When the hair color of photographs was assigned according to the enhanced method, the following hair colors were observed: sixty-one black, twenty-four dark brown/black, two brown/dark brown, and one blond/dark brown were observed. No brown, blond, and red categories were observed. EMC web tool predicted 59 black hair, 25 dark brown/black hair, and 4 brown/dark brown hair, with an accuracy of 78.6, 54.2, and 50% of accuracy, respectively (Table 3). 

When the hair colors of photographs were assigned employing the original HIrisPlex categories, 66 black (75%) and 22 brown (25%) hairs were observed in the population of Monterrey City. No blond or red hair colors were observed. For hair coloration, the variation of the results among predictors was greater than those obtained for eye color (Table 2). For the unrestricted data, UAS predicted seventy black and six brown hairs, of which fifty-seven and three were correct, respectively (accuracy = 68.2%).

On the other hand, EMC predicted fifty-four black and nineteen brown hairs, with 46 and 10 correct predictions, respectively (accuracy = 63.6%). When we filtered the results to those with probabilities >70%, prediction accuracy improved for both systems (UAS = 84%; EMC = 91%).

Interestingly, it is evident that better results were obtained with both predictors when the hair color was black, as both systems failed to predict brown hair with probabilities > 70%. It appears that UAS is more robust to predict hair coloration than EMC, when the *p* > 70% constraint is applied (42 vs. 20) (Table 2). Nevertheless, these comparisons were performed when the UAS color categories were employed (black, brown, blond, and red) and the black and brown hair color shades were omitted, which could cause a result bias in favor of UAS performance. Figure 1b shows three mismatch cases of prediction when the hair color was black. In the first case, both systems failed to assign one hair color but provided almost the same probability to brown and blond hair colors. In the second case, probabilities were 0.62 and 0.73 for the brown color in UAS and EMC, respectively. In the third case, UAS correctly predicted black color (*p* = 0.6), whereas EMC predicted brown color (*p* = 0.49) (Figure 1b).

## 4. Discussion

Although different studies have reported the prediction of externally visible characteristics in worldwide populations [21,29,30,31,32], the hair and eye color prediction based on the ForenSeq system has been scarcely reported. In the Americas, we only found two reports based on this forensic genomic system in US populations [22,23]. Therefore, the usefulness of this technology for admixed populations should be analyzed in depth. We report—for the first time in one Latin American population—the performance of the ForenSeq DNA Signature Prep kit to predict hair and eye coloration, specifically in Mexican Mestizos from Monterrey City.

### 4.1. Data Quality

As opposed to the EMC web tool, UAS is not able to provide phenotype estimations when a specific allele drop-out is present because it significantly affects the phenotype prediction [33,34]. Nevertheless, the 88 samples reported herein provided confident phenotype estimations with both predicting tools since the sequencing runs did not show significant allele drop-out (Supplementary Materials S1).

### 4.2. Population Characteristics

Mexican Mestizo populations are characterized by the presence of European, Native American, and African ancestries whose admixture processes took place since the European contact with the Americas around 500 years ago. However, the admixture pattern is not homogeneous throughout the country: northern populations show higher European ancestry while southern populations show more Native American ancestry; in general, the ancestry of Mexican Mestizo populations is related to the pre-Hispanic demography and recapitulates the Native American substructure [24,35]. Monterrey City is located in the northeast of Mexico, presenting one of the highest European ancestries in the country [24,36]. Interestingly, the origin of European conquers was principally Spain, and to a lesser extent Portugal and Italy [37]. Although Spain has a high frequency of blue and green/intermediate eyes [25], most of the studied population sample had brown eyes (85/88), and only three showed intermediate eye colors, one sample for each: green, hazelnut, and amber (Table 2, Figure 1a). Conversely, a relatively high frequency of blue and green eye color was reported in one Argentinean population sample [29]. Regarding hair color, we observed only brown and black related-shades. This is in line with a previous description from the Latin American population where blond hair was scarce and red hair was not observed [38].

### 4.3. Eye Color Prediction

The Monterrey City population showed 96.6% of brown eyes and 3.4% of intermediate eyes, whereas blue eyes were not found. This eye color distribution is in line with the data obtained based on the HGDP-CEPH Human Genome Diversity Cell Line Panel for Mexican populations [25]. Predictions obtained with both UAS and EMC systems were high with the pMax approach (accuracy = 96.6%) but improved with the *p* > 70% threshold (accuracy = 98.8%). Nevertheless, some considerations must be taken into account, for example, only brown eyes were correctly inferred by both systems, but the three cases of intermediate eye color assignments were incorrect. Although previous studies in European populations show high performance of the IrisPlex based-methods, in admixed populations, the blue eye color usually is scarce [25], with some exceptions such as the Argentineans [29]. We observed two eye colors but predominating brown color eyes; this skew in the color distribution causes an upward shift in the accuracy of the prediction, especially for those methods biased to the most frequent category that seems to offer better performance than they actually are [38]. Consequently, the high performance in eye color prediction seen in the Monterrey population could be biased by the brown eye color predominance, reflecting a better performance of this system to predict extreme brown and blue eye colors [4].

Although it was formerly thought that human eye color is a simple Mendelian phenotype [39], subsequent studies reported that it is a complex pattern of inheritance regulated by complex processes such as epistasis and incomplete dominance [40]. This could be one reason why the six SNPs included in the IrisPlex prediction-based methods show a low accuracy in the prediction of intermediate eye color, as was shown in our study in the Monterrey population, as well as in previous worldwide populations [21,29,30,31,33]. These findings highlight the necessity to include a greater number of loci in eye color prediction systems to better predict the intermediate eye color, especially for populations with high frequencies for this phenotype.

### 4.4. Hair Color Prediction

We observed a limited variety of hair colors (black, dark brown/black, brown/dark brown and blond/dark brown), with a predominance of black and dark brown/black shades. Blond and red hair colors were not observed in the Monterrey City population. This result is in line with a previous report on Latin American populations [38]. For Hirisplex, a prediction efficacy of 81–93% was initially reported for red, blond, brown, and black hair colors [41]. When we compared the predictions offered by UAS and EMC, employing the original hair colors of HIrisPlex, we obtained an accurate prediction of 68.2 and 63.6% based on pMax approach, respectively.

Although European populations showed better prediction results, they also show higher diversity in hair colors (black, brown, blond, and red hair) [21,30]. For both UAS and EMC, the black hair color was better predicted than brown, as previously described [21,30,33,38]. Thus, the low accuracy of hair prediction observed herein must be influenced by the presence of 25% of brown hair color. On the other hand, the low prediction of blond and brown hair colors may be influenced by the hair darkening that occurs in blond individuals from six to thirteen years old [42].

When we assessed the prediction results based on the EMC-enhanced method that considers the hair shade, a better prediction performance was observed (71.5%). Although this result is lower than the one obtained with the >70% threshold, the EMC-enhanced method allows hair color prediction without excluding samples with low probabilities values.

### 4.5. Predictor’s Performance

Both UAS and EMC showed the same prediction eye color performance, even employing the threshold criteria, as well as inconclusive results (*p* = ~0.30 for each color) in only one sample. When the shade was omitted for the hair color prediction, a higher number of inconclusive results were observed in the comparative analysis for both predictors (UAS = 11, EMC = 14), while no inconclusive predictions were observed when we applied the *p* > 70% threshold. Nevertheless, these accuracy results must be taken carefully. For instance, when we used the pMax approach, UAS showed 17 incorrect predictions (68.2% of accuracy) while EMC showed 18 incorrect predictions (63.6% of accuracy). Conversely, when the threshold was employed, UAS and EMC showed eight and two incorrect predictions, respectively, without inconclusive results. Although this seems good, a high number of predictions were excluded (UAS = 38 and EMC = 66) due to probabilities below the threshold (*p* < 70%). Thus, the higher accuracy predictions of the threshold (UAS = 84% and EMC = 91%) diminish considerably the number of samples (50 for UAS and 22 for EMC) because many are excluded.

Overall, both UAS and EMC had a similar accuracy precision for eye coloration, but for hair color prediction, UAS seems to be more accurate than EMC, both employing the pMax approach as well as the threshold. Conversely, previous studies in European populations showed a better performance of EMC for black and brown color hair, than those obtained by UAS [21]. This finding could be explained because in these studies, the enhanced method was employed; in fact, we obtained similar results with the enhanced method (accuracy of 71.5%). Moreover, the performance of hair prediction in Monterrey City could be biased by the almost exclusive presence of black hair color [38]. The same results will most likely apply to most of the Mexican Mestizo populations characterized by high Native American ancestry [24,35,36], which will be useful to evaluate future eye color prediction systems specifically designed to improve the prediction of intermediate eye colors. Finally, our prediction performance comparison in the Mexican population allows the recommendation of the enhanced method to predict hair color with the EMC web tool, instead of the threshold or regular pMax methods, which have the disadvantage of excluding samples from the prediction analysis.

## 5. Conclusions

We evaluated the 24 piSNPs included in the ForenSeq DNA Signature Prep kit to predict eye and hair color in Mexican Mestizos from the Monterrey City population. To our knowledge, this is the first report that predicts eye and hair color based on this forensic MPS technology in one Latin American population. Based on the UAS and EMC platforms, we observed a high-accuracy prediction for brown eye color. However, hair color prediction had a lower performance than in previous studies. Although employing a threshold of *p* > 70% improved the prediction accuracy, this causes the exclusion of a high number of results below the threshold; hence, the better option in forensic casework should be the pMax enhanced method, considering hair shade. Our results enable the reliable forensic use of this genomic platform to predict eye and hair color in this Mexican Mestizo population, principally in brown eyes and black hair colors, but with caution to predict hair phenotypes which are different from black hair. Future studies must be conducted with higher sample sizes in Mexican populations to evaluate the prediction performance of other possible eye and hair colors not observed in this study.

## Figures and Tables

**Table 1:** Allele frequencies, heterozygosity and Hardy–Weinberg evaluation of the 24 piSNPs studied in the Mexican Mestizo population of Monterrey City (*n* = 88).

Gene	SNP	Allele Frequencies				Ho	He	HWE *p*-Value
		A	T	G	C			
*SLC45A2*	rs28777	0.3636	-	-	0.6364	0.4318	0.4655	0.3663
*IRF4*	rs12203592	-	0.0284	-	0.9716	0.0341	0.0555	0.0584
*LOC105374875*	rs4959270	0.4830	-	-	0.5171	0.4205	0.5023	0.1428
*TYRP1*	rs683	0.4546	-	-	0.5455	0.4773	0.4987	0.8225
*TYR*	rs1042602	0.2273	-	-	0.7727	0.2500	0.3532	0.0100 *
*TYR*	rs1393350	0.1023	-	0.8977	-	0.1818	0.1847	1
*KITLG*	rs12821256	-	0.9943	-	0.0057	0.0114	0.0114	1
*LOC105370627*	rs12896399	-	0.2841	0.7159	-	0.3864	0.4091	0.6025
*SLC24A4*	rs2402130	0.8409	-	0.1591	-	0.2500	0.2691	0.4478
*OCA2*	rs1800407	0.0398	-	0.9602	-	0.0568	0.0768	0.0047 *
*MC1R*	rs312262906		-	-	1.0000	0	0	1
*MC1R*	rs1805005	-	0.0739	0.9261	-	0.1250	0.1376	0.3759
*MC1R*	rs1805006	-	-	-	1.0000	0	0	1
*MC1R*	rs2228479	0.0341	-	0.9659	-	0.0682	0.0662	1
*MC1R*	rs11547464	-	-	1.0000	-	0	0	1
*MC1R*	rs1805007	-	0.0114	-	0.9886	0.0227	0.0226	1
*MC1R*	rs201326893		-	-	1.0000	0	0	1
*MC1R*	rs1110400	-	0.9886	-	0.0114	0.0227	0.0226	1
*MC1R*	rs1805008	-	-	-	1.0000	0	0	1
*MC1R*	rs885479	0.3864	-	0.6136	-	0.5227	0.4769	0.4991
*TUBB3*	rs1805009	-	-	0.9886	0.0114	0.0227	0.0226	1
*PIGU*	rs2378249	0.9489	-	0.0511	-	0.1023	0.0976	1
*SLC45A2*	rs16891982	-	-	0.3466	0.6534	0.4432	0.4555	0.8178
*HERC2*	rs12913832	0.8693	-	0.1307	-	0.2386	0.2285	1

* SNPs out of Hardy–Weinberg expectations (*p* > 0.05); in grey are highlighted the cells of genes that predict eye color.

**Table 2:** Predictive results and accuracy of (**a**) eye and (**b**) hair coloration in Mexican Mestizos from Monterrey City using the ForenSeq™ DNA Signature Prep.

(a) Eye Color Prediction					
		Without Restriction Threshold		Threshold > 70%	
Color	Observed Color	UAS Prediction (CA)	EMC Prediction (CA)	UAS Prediction (CA)	EMC Prediction (CA)
Brown	85	86 (85)	86 (85)	85 (84)	85 (84)
Intermediate	3	0	0	0	0
Blue	0	1 (0)	1 (0)	1 (0)	0
Inconclusive	-	1	1	0	0
Excluded	-	0	0	3	3
Correct/ Incorrect	-	85/2	85/2	84/1	84/1
Percentage accuracy	-	96.6%	96.6%	98.8%	98.8%
**(b) Hair Color Prediction**					
		** Without Restriction Threshold **		** Threshold > 70% **	
**Color**	**Observed** **Color**	** UAS Prediction ** **(CA)**	** EMC Prediction ** **(CA)**	** UAS Prediction ** **(CA)**	**EMC Prediction (CA)**
Black	66	70 (57)	54 (46)	50 (42)	21 (20)
Brown	22	6 (3)	19 (10)	0	1 (0)
Blond	0	0	0	0	0
Red	0	0	0	0	0
Inconclusive *	-	11	14	0	0
Excluded **	-	0	0	38	66
Correct/ Incorrect	-	60/17	56/18	42/8	20/2
Percentage accuracy ***	-	68.2%	63.6%	84%	91%

CA: Correctly assigned; * Inconclusive: prediction results with <10% of difference between the higher probabilities; ** Excluded: samples excluded due to prediction probabilities < 70%; *** Percentage accuracy: taking into account the total number of predictions for this calculus in both UAS and EMC for each threshold.

**Table 3:** Hair color prediction results based on the enhanced model of EMC web tool.

Color	Observed Color	EMC Prediction (CA)	Accuracy (%)
Black	61	59 (48)	78.6
D. Brown/Black	24	25 (13)	54.2
Brown	0	0	-
Brown/D. Brown	2	4 (2)	50
Blond/D. Brown	1	0	0
Blond	0	0	-
Red	0	0	-
Correct/Incorrect	-	63/25	-
Percentage accuracy *	-	71.5%	-

CA, Correctly assigned. * Percentage accuracy: taking into account the total number of predictions for this calculus in both UAS and EMC for each threshold.

## Data Availability

The online version contains Supplementary Materials available on the journal webpage.

## Supplementary Materials

The following supporting information can be downloaded at: https://www.mdpi.com/article/10.3390/genes14051120/s1, Supplementary Material S1: SNP genotype database of the studied Mexican Mestizo population sample (*n* = 88). Supplementary Material S2: Results of the pairwise LD tests (*p*-values) between piSNPs studied in the Mexican Mestizo population sample (*n* = 88).
